# Lung Transplant Recipients and COVID-19: Report of Two Cases

**DOI:** 10.3390/jcm12134287

**Published:** 2023-06-26

**Authors:** Filippo Antonacci, Matteo Petroncini, Elena Salvaterra, Pietro Bertoglio, Niccolò Daddi, Giulia Lai, Jury Brandolini, Piergiorgio Solli, Giampiero Dolci

**Affiliations:** 1 Thoracic Surgery, IRCCS Azienda Ospedaliero-Universitaria di Bologna, 40138 Bologna, Italy; matteo.petroncini@gmail.com (M.P.); pietro.bertoglio@aosp.bo.it (P.B.); niccolo.daddi@unibo.it (N.D.); giulia.lai@aosp.bo.it (G.L.); jury.brandolini@ausl.bologna.it (J.B.); piergiorgio.solli@ausl.bologna.it (P.S.); giampiero.dolci@aosp.bo.it (G.D.); 2 Interventional Pulmonology, IRCCS Azienda Ospedaliero-Universitaria di Bologna, 40138 Bologna, Italy; elena.salvaterra@aosp.bo.it

**Keywords:** lung transplant, COVID-19, chronic lung allograft rejection

## Abstract

Although the WHO has declared the end of the pandemic emergency, COVID-19 still poses a threat to immunocompromised patients. The COVID-19 pandemic has spread throughout the world over the last two years, causing a significant number of deaths. After three years, SARS-CoV-2 has lost its initial lethality but has shown a significantly worse prognosis for immunocompromised patients, especially those who have undergone lung transplantation, compared with the general population. This paper presents two compelling case studies that highlight the complex challenges of COVID-19 infection in lung transplant recipients. The first case involves a patient who received a bilateral lung transplant for pulmonary artery hypertension in 2009, followed by a kidney transplant in 2022. Surprisingly, despite an initially favorable clinical course after contracting COVID-19, the patient deteriorated rapidly and died within a few days due to extensive lung involvement. This case highlights the unpredictable nature of COVID-19 and its potentially devastating impact on lung transplant recipients. The second case involves a patient who underwent bilateral lung transplantation five years earlier for chronic obstructive pulmonary disease (COPD). This individual also contracted COVID-19 and had pre-existing complications, including chronic lung allograft rejection (CLAD) and diffuse bronchial stenosis. Following viral infection, the patient’s clinical condition deteriorated rapidly, with worsening bronchial stenosis. This case highlights the ability of COVID-19 to exacerbate pre-existing pulmonary complications in transplant recipients. These cases highlight the urgent need for increased vigilance and tailored management strategies when dealing with COVID-19 in lung transplant recipients. The unpredictable and detrimental course of the disease observed in these patients highlights the importance of implementing stringent preventive measures, such as vaccination and strict adherence to infection control protocols, in this vulnerable population. Further research is essential to gain a full understanding of the unique dynamics of COVID-19 in lung transplant recipients and to develop targeted interventions to improve their outcomes.

## 1. Introduction

The World Health Organization (WHO) declared the end of the pandemic emergency on 5 May 2023, but COVID-19 is far from being defeated, especially when it comes to vulnerable populations such as solid organ transplant recipients. Based on information from the Centers for Disease Control and Prevention, COVID-19 commonly presents with symptoms such as fever, cough, shortness of breath, muscle aches and, in some cases, loss of taste or smell. Gastrointestinal symptoms may also occur, including nausea, vomiting, diarrhea and abnormal liver function. Less common manifestations include headache, dizziness, conjunctivitis, eye irritation and a rash characterized by redness of the skin [1]. In recent years, several retrospective studies have focused on analyzing the outcomes, clinical features and management of COVID-19 in vulnerable patient populations, including those who have undergone allogeneic stem cell transplantation (allo SCT) and lung transplantation (LT) [2,3]. These studies provide valuable insights into the management and care of these specific patient populations. A study conducted in German and Austrian transplant centers aimed to analyze the outcomes of COVID-19 in allo SCT recipients. The cohort included 130 patients who had previously undergone allo SCT who then contracted SARS-CoV-2 between February 2020 and July 2021. The median age at COVID-19 diagnosis was 59 years, and the median age at allo SCT was 55 years. Common underlying diseases among patients included acute myeloid leukemia, Hodgkin’s and non-Hodgkin’s lymphoma, acute lymphoblastic leukemia and myelodysplastic syndrome. Many patients were in incomplete remission at the time of their COVID-19 diagnosis and had active graft versus host disease (GVHD) requiring systemic immunosuppressive treatment. COVID-19 detection methods varied, with nasopharyngeal swab Polymerase Chain Reaction (PCR) being the most common. Transmission was mainly within the family and home environment. Symptoms such as fever, cough, dyspnea and fatigue were common, and a significant proportion of patients developed pneumonia. Around 19% of patients required intensive care, and some patients received specific treatments such as corticosteroids, convalescent plasma, remdesivir and bamlanivimab. This study aimed to identify risk factors for severe disease and mortality in allo SCT recipients with COVID-19, highlighting the need for a comprehensive understanding of the impact of COVID-19 on this patient population to guide appropriate management strategies and to optimize patient care. Similarly, a retrospective, multicenter cohort study conducted in French lung transplant centers had investigated the clinical characteristics and outcomes of LT recipients with COVID-19 [4]. The study included 35 LT recipients with confirmed or highly suspected SARS-CoV-2 infection. Double lung transplants were the most common, with a median time to LT of 38.2 months. Immunosuppressive therapies, including calcineurin inhibitors and corticosteroids, were commonly used in these patients.

Clinical presentation was variable, with fever being the predominant symptom. Chest CT scans typically showed ground-glass opacities. The study reported cases of hospital-acquired, healthcare-associated and community-acquired infections. Management strategies included the adjustment of immunosuppressive therapy and the administration of specific antiviral treatments such as hydroxychloroquine and remdesivir. The results of the study showed an overall survival rate of 85.7% after a median follow-up of 50 days. However, five patients died due to multi-organ failure and acute respiratory distress syndrome (ARDS). Thrombotic events and pulmonary superinfections were observed in some patients. Overweight status was identified as a potential risk factor for death in LT recipients with COVID-19, while no other significant risk factors for severe disease or mortality were found. In addition, ongoing research and studies are being conducted to assess the effectiveness and safety of COVID-19 vaccination in lung transplant recipients, providing valuable insights and guidance for their healthcare management. COVID-19 vaccination has been a crucial tool in fighting the global pandemic. With the development and distribution of effective vaccines, it offers hope for a safer and healthier future. Vaccines help stimulate the immune system to recognize and fight off the virus, reducing the severity of symptoms and preventing hospitalizations and deaths. However, for individuals who have undergone lung transplants, the importance of vaccination is even more significant. Transplant recipients typically have a weakened immune system, making them more vulnerable to infections. Getting vaccinated against COVID-19 can provide an additional layer of protection and minimize the risk of severe illness or complications in this vulnerable population. It is essential for transplant recipients to consult their healthcare providers for specific vaccination guidelines and recommendations. These studies contribute to our understanding of the clinical characteristics and outcomes of COVID-19 in allo SCT and LT recipients and highlight the importance of tailored management strategies for this vulnerable population. Further research is needed to gain a comprehensive understanding of the risk factors and optimal management approaches for COVID-19 in these patient groups.

## 2. Case #1

**Figure 1:**
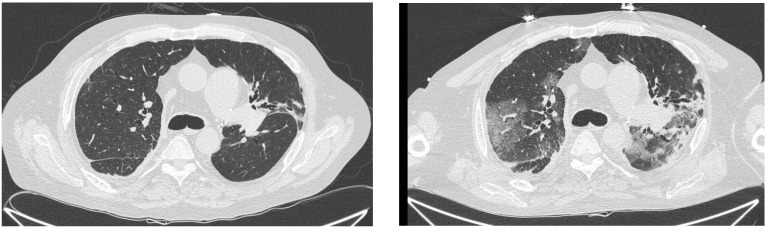
Case #1. Chest CT in December 2022 (**left**) and January 2023 (**right**). In January 2023, we observed an increased lung parenchymal involvement compared with December 2022.

The first patient who developed COVID-19 was a 58-year-old man with a history of ischemic heart disease with normal cardiac function, osteoporosis and obesity. He underwent bilateral lung transplantation in 2009 for idiopathic pulmonary hypertension using intraoperative veno-arterial Extra Corporeal Membrane Oxygenation (ECMO). He did not need prolonged ECMO support in the intensive care unit and remained on mechanical ventilation for 17 days. He did not develop any serious early postoperative complications except for lobar pneumonia, which was treated with targeted antimicrobial therapy, and atrial fibrillation, requiring pharmacological cardioversion. He was discharged home on postoperative day 47. Following the long-term use of calcineurin inhibitors, he developed chronic renal failure, which rapidly progressed to severe renal insufficiency requiring peritoneal dialysis. He was a kidney transplant candidate and underwent a kidney transplant on 15 October 2022. He did not develop a SARS-CoV-2 antibody response despite receiving three doses of an mRNA anti COVID-19 vaccine at the start of the pandemic vaccination campaign after meeting the frailty criteria. After a positive COVID-19 serological test two months prior to kidney transplantation, he also underwent treatment with tixagevimab/cilgavimab. In this case, the disease remained asymptomatic. After the kidney transplant, his renal function was suboptimal, with a mean creatinine of 2 mg/dL (normal <1.2 mg/dL). His lung function remained suboptimal after the kidney transplant, but the last follow-up endoscopic biopsy showed an early chronic allograft rejection-like pattern. On 8 December 2022, the patient presented with mild upper respiratory symptoms and tested positive for COVID-19 by an antigen swab test. The patient’s immunosuppressive regimen at that time included corticosteroids, calcineurin inhibitors and antimetabolites. Molnupiravir was promptly administered with no change in immunosuppressive therapy, and his clinical course remained stable with a mild symptomatic syndrome. Twenty days after the initial positive test, the patient presented at the emergency department with shortness of breath and cough. A chest CT scan showed mild infiltrates bilaterally and a stable volume of right pleural effusion. Due to the persistence of a positive antigen swab test and the chest CT findings, the patient was admitted to the hospital for observation. After five days, he was discharged in a stable clinical condition, with no respiratory failure and with stable renal function. The immunosuppressants were continued as prescribed. However, the patient’s condition subsequently deteriorated, with progressive respiratory failure and increased lung infiltrates observed on follow-up chest CT scans. As a result, on 7 January 2023, he was readmitted to our hospital lung transplant unit, where he was isolated in an intensive care unit box with a diagnosis of ARDS. Figure 1 shows how his lung parenchymal involvement worsened between December 2022 and January 2023. The medical team decided to withhold calcineurin inhibitors and antimetabolites to treat the severe COVID-19 infection. Piperacillin/tazobactam, a broad-spectrum antibiotic, was started, but unfortunately, there was no improvement in the chest CT findings and the patient’s gas-exchange function progressively deteriorated. Subsequent chest X-rays showed an increased number of pulmonary infiltrates. A last attempt to treat the patient with a trial of the antiviral drug remdesivir was considered. However, despite these efforts, the patient’s condition continued to deteriorate, and he died on 19 January 2023.

## 3. Case #2

**Figure 2:**
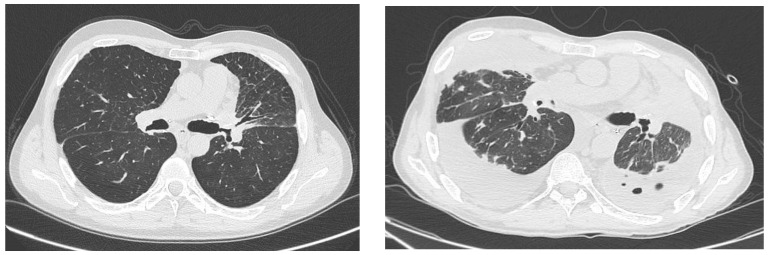
Case #2. Chest CT in March 2021 (**left**) and February 2023 (**right**). The lung parenchymal involvement worsened in February 2023, compared with March 2021, with the development of pneumonia and pleural effusion.

We have observed a second peculiar case of COVID-19 in lung transplant recipients in our center. It involved a 52-year-old man who underwent bilateral lung transplantation in 2017 for panlobular and centrilobular emphysema due to alpha 1 antitrypsin deficiency. During the transplant, he was supported by ECMO via aorto-atrial cannulation. Post-transplantation, he did not require ECMO prolongation and was extubated on post-operative day 2. After 5 days, he was discharged from the ICU with good respiratory function. His length of stay was 16 days. Six days after being discharged home, he attended the outpatient clinic, and subcutaneous emphysema was present. An urgent CT scan was performed immediately and showed moderate pneumomediastinum, pneumothorax and pneumopericardium. Bronchoscopy showed a small dehiscence of the left bronchial anastomosis. He was rapidly referred to the Interventional Pulmonology Unit, where he was treated with an endoscopic application of fibrin glue and discharged home. However, after three months, the patient developed severe bronchial stenosis in the left main bronchus. This condition was treated several times with laser and mechanical dilatation, but with only partial success, leaving a residual lumen of 5 mm. The patient subsequently developed significant stenosis in the left upper lobe and right upper and middle lobe bronchi. He continued to be followed up at our lung transplant center. During this period, he developed stenosis due to malacic degeneration of the lower third of the trachea, stenosis of the right lobar bronchus and subtotal occlusion of the left upper lobar bronchus with atelectasis of the left upper lobe, as shown on the March 2021 CT scan. In Figure 2, the CT scan, performed in March 2021, showed the presence of bronchial stenosis without evidence of pneumonia (Figure 2). A bronchial biopsy performed during follow-up showed early chronic lung allograft dysfunction, and cultures were positive for Pseudomonas aeruginosa. Therefore, the immunosuppressive therapy consisting of corticosteroids, calcineurin inhibitors and antimetabolites was modulated and he received antibiotic therapy to eradicate the bacterial colonization. The patient had received three doses of mRNA anti COVID-19 vaccine and showed an antibody response. His respiratory function was mildly impaired, with a forced expiratory volume in one second (FEV1) of 35% and a forced vital capacity (FVC) of 45%, but this remained stable over time. No desaturation was observed during the 6-minute walk test. On 5 July 2022, the patient tested positive for COVID-19 and presented with persistent cough and shortness of breath which was treated with nirmatrelvir and ritonavir. Again, the immunosuppressive regimen of corticosteroids, calcineurin inhibitors and antimetabolites was not changed. In July 2022, after the patient tested negative for COVID-19, he presented at our center for a routine outpatient evaluation with significantly worsened respiratory function with an FEV1 of 30% and an FEV1/FVC ratio of 45%. The chest CT scan showed a bronchitis-like radiological pattern with oedema of the left main bronchus and a left pneumothorax, which was considered a COVID-19-related evolution of the pre-existing partial atelectasis reported on the previous CT scan. Urgent admission to hospital was arranged for a repeat bronchoscopy and possible lobectomy. Endoscopy revealed an unfavorable progression of the bilateral bronchial stenosis, and the patient experienced a significant clinical worsening. Given the futility of both endoscopic (bronchial balloon dilation) and surgical (lobectomy of atelectatic lobe) treatment and the rapid and significant decline in lung function following the infection, he was assessed for retransplantation. He was discharged after normalization of his inflammatory markers and 92% oxygen saturation was achieved after daily respiratory physiotherapy. However, due to a further deterioration in his respiratory status, the patient was re-admitted to hospital in February 2023 and died a few days later from left-sided pneumonia resulting in untreatable type-2 respiratory failure. From Figure 2, it is evident how the lung parenchymal involvement worsened, with the development of pneumonia and pleural effusion.

## 4. Discussion

COVID-19 was a global challenge for the entire scientific community. However, over time, improved knowledge of the physiopathology of the virus has led to more successful treatments. Currently, we need to be aware of the potential threat that COVID-19 poses to frail patients. Solid-organ recipients are highly susceptible to developing severe forms of COVID-19 due to their chronic immunosuppression. This risk is even higher in lung transplant recipients, who have a higher degree of immunosuppression, a high incidence of allograft rejection and exposure of the graft to the external environment [5,6]. Although the majority of lung transplants performed in our center in the last four years were for pulmonary hypertension, the cases presented in this paper are related not only to vascular lung pathology but also to parenchymal disease, such as emphysema. This is to highlight the different initial conditions that led the two patients to these unusual scenarios. The most interesting finding, in our opinion, is the completely different evolution of the disease, in terms of radiological and clinical findings, as well as the timing of their progression. The second important point are the different treatments adopted: in the first case, medical treatment was modified, whereas in the second case, we initially opted for endoscopic bronchial dilatation or surgical resection (which was not eventually performed given the futility of the procedure) and subsequently for listing for retransplantation.

The last evidence resulting from our cases regards the identification of possible risk factors: immunosuppressive therapy, CLAD and bronchial stenosis.

As described in non-immunocompromised hosts, the clinical presentation of COVID-19 in transplant patients is mostly characterized by fever and cough, especially after the introduction of anti-COVID-19 vaccines [4]. Our cohort of patients confirms this trend: out of 60 lung transplant patients followed by our unit, 30 developed COVID-19 infection, but only two patients developed a severe form of the infection. However, it is important to note that this series of patients has not been published.

The radiological findings are characterized by the presence of ground-glass opacities on CT, as described in the general population [4]. Ground-glass opacities are also the main radiological findings of COVID-19 in lung transplant recipients, but radiological presentation may vary among individuals and can be influenced by factors such as the timing of the scan, disease severity, and the presence of pre-existing lung conditions or anatomical changes related to the transplant. The two reported cases have unique features. In particular, Case #1, interestingly, developed a clinically relevant form of COVID-19 one month after testing positive for the virus. Despite treatment with molnupiravir, the patient’s respiratory status deteriorated dramatically 30 days after infection. The recent kidney transplant probably played a critical role in further weakening the patient’s immune system and making him more susceptible to COVID-19. According to Raja et al., lung transplant recipients, and solid-organ recipients in general, show longer viral clearance of SARS-CoV-2 than the general population—almost 3–5 weeks. T-cell immunity, which is pharmacologically inhibited after transplantation, is thought to be the first actor in the antiviral response [7]. On the other hand, Opsomer et al. reviewed 77 articles and did not find any association between the type of immunosuppression and mortality [8]. They also found a less relevant immune response after the first two doses of vaccination. These findings are corroborated by Altneu et al., who also support the need for a fourth dose in recipients, especially those infected with the Omicron variant [9]. In Case #2, the patient was already suffering from significant bilateral bronchial stenosis, and COVID-19 contributed to accelerating the process of respiratory deterioration. In our opinion, Case #1 has a rather peculiar aspect, as the late onset of COVID-19-related pneumonia in the immunosuppressed patient has never been described in lung transplant recipients. Therefore, we believe that these two case reports represent opposite forms of COVID-19 presentation in lung transplant recipients. In the first case, we saw a late-onset ARDS due to parenchymal involvement. In the second case, the pathophysiological mechanism is more related to the involvement of large bronchial structures. This highlights some important aspects.

The issue that is more discussed in literature is the extreme frailty of these patients, not only in the acute phase but also one month after infection.

In COVID-19 infection, the treatment strategy includes the withdrawal of antimetabolites and an increase in corticosteroids, with or without withdrawal of calcineurin inhibitors. Treatment of the infection consists of specific antiviral drugs (remdesivir or lopinavir–ritonavir) [4]. The treatment for Case #1 was exclusively based on medical therapy. There are no specific guidelines for the timing for antimetabolite withdrawal, but we could hypnotize that a late modification of therapy may be the reason for the severe pneumonia onset. Modification of immunosuppressive therapy probably played an important role in the progression of the parenchymal involvement in Case #1. The French Transplant Society has suggested that the treatment strategy should be based on the severity of the disease [4]. In this latter study, the most common change in immunosuppressive therapy was also the withdrawal of antimetabolites, followed by an increase in the corticosteroid dose [4]. However, no definitive indication can be given, as each transplant center has adopted a strategy tailored to the clinical characteristics of the patient.

On the other hand, in Case #2, we observed the worsening of bronchial stenosis. In this specific case, the medical treatment was unsuccessful, and we resorted to surgical solutions without the possibility of success. There were no similar cases in the literature to compare with.

These cases highlight potential prognostic factors in lung transplant recipients who develop COVID-19-related disease. The mortality associated with COVID-19 in lung transplant recipients reported in the literature ranges from 10% to 46%. In our cohort of lung transplant recipients, 30 out of 60 patients developed COVID-19, reporting a mortality of 6.6% (2/30 patients). This cohort of patients has never been published. We propose two causes to explain these data. First, in Italy, we observed a high level of compliance with the COVID-19 restrictions (isolation, use of masks, etc.), thus limiting the rate of infection, even in our transplant population as a whole, during the first phase of the pandemic, when no vaccines were available. Second, the majority of COVID-19 infections in our population were observed during the second wave, when the population was already widely vaccinated, and the clinical manifestations of infection were therefore milder.

Regarding long-term follow-up after COVID-19 infection, it has been shown that the FEV1 remained stable, whereas the TLC and DLCO decreased significantly due to the interstitial involvement and restrictive pattern resulting from the infection [4]. Kamp et al. showed that the burden of comorbidities, as assessed by the Charlson Comorbidity Index, was a predictor of poor outcome in this group of patients [5]. In addition, Messika et al. reported that overweight status (body mass index >25 and <30 kg/m^2^) was associated with an increased risk of death [4].

In CLAD, COVID-19 may play a crucial role in determining the clinical course in terms of death (43% of cases) or a further decline in the FEV1 (43% of cases), and it should be noted that none of these patients received any vaccination prior to infection. Messika et al. showed a lower mortality (14.3%) but, as reported by Kamp et al., their series had a lower rate of CLAD, a lower median age and BMI and a shorter observation period [4,5], while Permpalung et al. reported no significant association between COVID-19 and the possible worsening of pre-existing CLAD [10]. These cases show that we must be aware of the possible long-term ineffectiveness of preventive measures and acute treatments such as vaccines, social distancing and antiviral therapy. Indeed, all preventive measures may be ineffective when combined with the frailty of lung transplant recipients.

Finally, the interaction between viral injury and post-transplant anatomical changes, such as bronchial stenosis, must never be underestimated. There is little evidence of bronchial changes after SARS-CoV-2 infection as in Case #1. However, Kanne et al. reported that bronchial thickening is a common complication of long COVID-19 [11]. Visconti et al., analyzing a Brazilian cohort of pulmonary COVID-19, found many late complications of infection. They reported an 18% rate of bronchial thickening two months after discharge as measured by CT scans [12].

In conclusion, the first case highlights the challenges faced by transplant recipients with COVID-19, particularly those with complex medical histories and immunosuppressive regimens.

The patient’s experience highlights the need for continuous monitoring, individualized treatment approaches and further research to improve the management and outcomes of COVID-19 in this vulnerable population. We also believe, based on the second case report, that in susceptible patients, SARS-CoV-2 may involve the bronchus and exacerbate stenosis or bronchiectasis. We also do not yet know the full spectrum of viral effects on lung anatomy, but it is reasonable to assume that COVID-19 infection played a primary role in the final occlusion of the bronchial tree in Case Report #2.

## 5. Conclusions

In conclusion, the COVID-19 pandemic has posed significant challenges for lung transplant recipients, but ongoing research and improved understanding of the virus will contribute to better management and outcomes. Lung transplant recipients are highly frail patients who, if affected by CLAD, require prompt evaluation and management if they test positive for COVID-19. The presence of transplant complications, whether anatomical (such as bronchial stenosis) or immunological (such as CLAD), must be considered a risk factor for poor prognosis when lung transplant recipients develop COVID-19. Immunosuppressive therapy plays a major role in the development of severe disease, and late pharmacological adjustment could be detrimental to the patient’s health. There is a lack of data on the transplant recipient population, and COVID-19 is a recent phenomenon with many unknown characteristics. Continued adherence to preventive measures, such as vaccination and social distancing, is crucial in protecting this vulnerable population. Further studies are needed to clarify the impact of SARS-CoV-2 infection in lung transplant recipients.
